# Items

**Published:** 1882-06

**Authors:** 


### Items.

THE TONGA TRADE MARK LITIGATION.

The suit of the English firm of Allen & Hanburys *v.* Parke Davis & Co., of Detroit, has abruptly terminated by the plaintiffs, on their own motion, obtaining an order of court to dismiss the bill of complaint and assuming the costs of suit themselves.

This affair has ended all too soon for the plucky Detroit firm, who hoped to place a decision on record in the United States court as to whether a firm has the right to appropriate and monopolize by trade mark the common name of an article, and thus exclude others from its manufacture, effectually destroying competition.

The compound known as Tonga having been introduced to the profession by such distinguished authority as Drs. Murrell, Ringer and others, Parke Davis & Co. cast about for a supply of the article for manufacture. Its habitat being in the Fiji Islands, some seven thousand miles southwest of San Francisco, a special agent sent by them secured in six months a large supply of the drug. In accordance with their custom, they published to the trade and medical profession what information they have been able to secure, and supplied to hospitals and many practitioners sample quantities for trial.

As a result of this enterprise, a demand sprang up for the drug, which attracted the notice of the English firm.

Supposing a simple warning sufficient to induce the American firm to relinquish the fruit of its labor and enterprise, they politely informed them that the name "Tonga" and the combination of drugs known by that name were the special property of Allen &

Hanburys under a registered trade mark, and that all trade in the article except through them should now cease.

Parke Davis & Co. refused to entertain any such demand, and the English firm, after failure to obtain letters patent in the United States, brought suit against them in the United States court for infringement of trade mark with the above result.

---

MORTALITY in the chief cities of the United States for the year 1881: Compiled from National Board of Health Bulletin.

| | Population. | Mortality per 1,000. |
|---|---|---|
| Portland, Me | 33,810 | 18.5 |
| Concord, N. H. | 13,838 | 17.9 |
| Burlington, Vt | 11,364 | 18.1 |
| Boston, Mass | 362,535 | 24.1 |
| Lowell, Mass | 59,485 | 21.6 |
| Worcester, Mass | 58,295 | 19.3 |
| Providence, R. I. | 104,857 | 20.2 |
| New Haven, Conn | 62,882 | 20.4 |
| New York, N. Y. | 1,206,577 | 31.9 |
| Brooklyn, N. Y. | 566,689 | 25.2 |
| Buffalo, N. Y. | 155,137 | 25.6 |
| Newark, N. J | 136,400 | 25.5 |
| Philadelphia, Pa | 846,980 | 28.6 |
| Pittsburgh, Pa | 156,381 | 28.6 |
| Wilmington, Del | 42,499 | 31.0 |
| Dist. Columbia | 180,000 | 24.8 |
| Richmond, Va | 63,803 | 32.1 |
| Wheeling, W. Va | 31,266 | 22.7 |
| Augusta Ga | 23,023 | 31.6 |
| Atlanta, Ga | 37,421 | 25.7 |
| Key West, Fla | 9,890 | 24.7 |
| Mobile, Ala | 31,205 | 31.0 |
| Vicksburg, Miss | 11,814 | 30.8 |
| New Orleans, La | 216,140 | 29.6 |
| Galveston, Tex | 22,253 | 30.9 |
| Nashville, Tenn | 43,461 | 26.4 |
| Louisville, Ky | 123,645 | 22.2 |
| Cincinnati, Ohio | 255,708 | 24.3 |
| Cleveland, Ohio | 160,140 | 23.5 |
| Indianapolis, Ind | 75,074 | 21.1 |
| Chicago, Ill | 503,304 | 27.2 |
| Milwaukee, Wis | 115,578 | 23.2 |
| St. Paul, Minn | 41,498 | 23.4 |
| E. Saginaw, Mich | 19,016 | 15.0 |
| Dubuque, Iowa | 22,254 | 12.5 |
| St. Louis, Mo | 350,000 | 23.9 |
| Omaha, Neb | 30,508 | 17.1 |
| Salt Lake City, Utah | 20,768 | 27.9 |
| San Francisco, Cal | 233,956 | 17.6 |
| Average | 24.7 | |

Mean death-rate from the following diseases per 1,000 :

| | |
|---|---|
| Accidents | 0.80 |
| Consumption | 3.23 |
| Croup | 0.50 |
| Diarrhœal diseases | 2.49 |
| Diphtheria | 1.01 |
| Typhoid fever | 0.65 |
| Scarlet fever | 0.67 |
| Pneumonia | 1.52 |
| Bronchitis, acute | 0.57 |
| Small-pox | 0.45 |

*Chicago.*—The registrar reports a total of 1,178 deaths during the month of January, of which there were 24 from croup; 46 from diphtheria; 13 from cerebro-spinal fever; 30 from scarlet fever; 62 from typhoid fever; 133 from small-pox; 120 from phthisis; 210 from acute lung diseases, etc.

---

RICORDIANA.—The French doctor is fond of flavoring his professional work and reading with literary digressions and witty sayings. A surgeon or physician to be really great in Paris must utter an occasional epigram. There are amusing anecdotes told of every prominent Parisian medical man. There exists a collection of these "morceaux choisis." To judge from the collection, Ricord is the greatest man in Paris. The book is full of stories about him. Here are some "Ricordiana:"

"Ricord is a savant whose researches have rectified an important point in mythology. He has demonstrated that it was not Vulcan, but Mercury whom Venus should marry."

"Some one asked Ricord if small-pox and the other were of the same family. 'They are sisters,' said he, 'but not of the same bed.'"

"'The condom,' says Ricord, 'is a cuirass against pleasure and a cob-web against danger.'"

"At the Convent of St. Jean de Latran there is preserved a famous relic in the shape of a serrated ring used by a mediæval saint for the discipline of the rebellious flesh. Ricord was one day asked by a young lady, an *ingenue de salon*, what was the character of this relic, made doubly famous by the witticisms of Voltaire. Ricord was somewhat embarrassed at first. 'Ah, yes;' replied he, 'it is a crown of thorns.'"

